# A Neural-Operator Surrogate for Platelet Deformation Across Capillary Numbers

**DOI:** 10.3390/bioengineering12090958

**Published:** 2025-09-06

**Authors:** Marco Laudato

**Affiliations:** FLOW Research Center, Department of Engineering Mechanics, KTH Royal Institute of Technology, SE-10044 Stockholm, Sweden; laudato@kth.se

**Keywords:** platelet, thrombosis, neural networks, DeepONet

## Abstract

Reliable multiscale models of thrombosis require platelet-scale fidelity at organ-scale cost, a gap that scientific machine learning has the potential to narrow. We trained a DeepONet surrogate on platelet dynamics generated with LAMMPS for platelets spanning ten elastic moduli and capillary numbers (0.07–0.77). The network takes as input the wall shear stress, bond stiffness, time, and initial particle coordinates and returns the full three-dimensional deformation of the membrane. Mean-squared-error minimization with Adam and adaptive learning-rate decay yields a median displacement error below 1%, a 90th percentile below 3%, and a worst case below 4% over the entire calibrated range while accelerating computation by four to five orders of magnitude. Leave-extremes-out retraining shows acceptable extrapolation: the held-out stiffest and most compliant platelets retain sub-3% median error and an 8% maximum. Error peaks coincide with transient membrane self-contact, suggesting improvements via graph neural trunks and physics-informed torque regularization. These results represent a first demonstration of how the surrogate has the potential for coupling with continuum CFD, enabling future platelet-resolved hemodynamic simulations in patient-specific geometries and opening new avenues for predictive thrombosis modeling.

## 1. Introduction

Scientific machine learning is challenging the long-standing computational barrier that separated molecular-scale platelet physics from organ-scale blood-flow models. Physics-informed [1] and operator-learning architectures (e.g., DeepONet [2]) can learn the nonlinear map between membrane-level mechanics and flow conditions directly from high-fidelity particle- or molecular-dynamics data and then inject that knowledge into a continuum computational fluid dynamics (CFD) solver at virtually zero computational cost. Recent platelet-specific implementations [3,4] show that a single DeepONet evaluation reproduces full platelet dynamics at molecular level with sub-percent error while accelerating the micro-solver by four to five orders of magnitude, potentially enabling simulations with clinically realistic platelet counts and geometries that were previously infeasible [5].

The application of such surrogates in a multi-scale model loop can be impactful for thrombosis modeling [6], where clot initiation depends on nanometer-scale activation [7] yet is modulated by vessel-scale hemodynamics [8]. Bridging these two complementary descriptions of thrombosis is crucial [9]. Continuum-mechanics models excel at capturing hemodynamics in both idealized [10,11,12] and complex, patient-specific geometries [13,14,15,16], making them indispensable for clinically relevant scenarios, while molecular-dynamics simulations deliver high-fidelity in the mechanochemical behavior of individual blood components, including receptor binding, membrane deformation, and chemical signaling [17,18,19].

By coupling operator-learned surrogates like DeepONet into a multiscale loop, it is possible to unify the geometric flexibility of continuum approaches with the atomistic accuracy of molecular models, thereby obtaining a holistic (and computationally tractable) framework for thrombus initiation and progression. The fidelity of such a multiscale framework is bounded by the surrogate’s ability to emulate the underlying molecular dynamics; rigorous benchmarking across the governing dimensionless groups is therefore mandatory. In this study we focus on the capillary number, $Ca^{*}$, that can be interpreted as the ratio of viscous to elastic forces [20] acting on the platelet. This number governs platelet and red-blood-cell deformation and margination in shear flows, as summarized in Table 1 and extensively characterized in prior work on blood-cell suspensions.

As Ca increases, platelets undergo tank-treading motions and flattening that enhance their lateral drift toward the vessel wall (margination), whereas at low Ca they retain a more rigid, discoidal shape with limited near-wall transport and adhesive surface area [25]. These deformation-driven changes also modulate platelet–red blood cell collision rates and shear-gradient diffusivity, both of which sensitively scale with $Ca^{*}$ and determine how frequently platelets encounter and adhere to the endothelium [26]. In thrombosis, where clot initiation is based on the interplay between shear-driven transport and receptor–ligand binding kinetics, capturing the full spectrum of $Ca^{*}$-dependent behaviors is essential. Systematic studies of $Ca^{*}$ therefore provide the mechanistic underpinning for training and validating neural-operator surrogates like DeepONet across the physiologically relevant deformation regimes encountered in vivo.

In this study, we quantify the accuracy of a DeepONet-based surrogate in capturing the time-dependent deformation of a simplified model of a platelet suspended in Couette flow. The platelet geometry is a 3D ellipsoid represented by network of roughly 20,000 particles linked by harmonic springs, with variations in the capillary number achieved by adjusting the spring constant. At each time step, the neural operator receives the current flow conditions and membrane parameters and returns the platelet’s instantaneous particle configuration. To assess the surrogate’s robustness, we systematically evaluated model performance across a range of capillary numbers, conducting both interpolation tests within the training regime and extrapolation tests beyond it. This framework allows us to determine the surrogate’s predictive limits and to establish guidelines for its deployment in multiscale thrombosis simulations.

The platelet’s particle-based model and the governing hemodynamics are presented in Section 2. In Section 3, we detail the neural operator architecture applied in this work. Finally, Section 4 discusses the surrogate’s error across varying capillary numbers.

## 2. Particle-Based Platelet Model

The learning dataset was generated with LAMMPS [27] by coupling dissipative particle dynamics (DPD) [28] for the surrounding blood flow to a spring-network representation of the platelet as a 3D hollow ellipsoid, following the workflow established in our previous studies. This simplified geometry was chosen deliberately as our objective in this work for methodological purposes: to demonstrate that a neural-operator surrogate can learn and reproduce the full, time-resolved deformation of a three-dimensional particle-based model and to quantify its accuracy across a broad range of capillary numbers. A discoid ellipsoid is sufficiently non-trivial to exhibit the canonical Jeffery-type rotation and tank-treading observed for quiescent platelets in shear flow, yet it keeps the cost of generating the $O(10^{6})$ training snapshots per trajectory tractable. In our initial work [3], we sampled 101 discrete shear stresses (50–250 Pa) but recorded only the end-state platelet shapes after one Jeffery orbit, whereas a subsequent study [4] resolved the full time evolution at a single shear stress (50 Pa). In the present work, we capture complete deformation trajectories across 10 different capillary numbers, obtained by varying the harmonic-bond stiffness. In total, 101 distinct time instants are sampled for the 10 values of the harmonic-bond elastic constant *K* (Table 2). The resulting dimensional capillary number

(1) $$Ca^{*}=\frac{\mu \;\dot{\gamma }\;a}{G_{s}}$$

covers the physiologically relevant range $0.07<Ca^{*}<0.7$, where μ is the blood viscosity, $\dot{\gamma }$ the imposed shear rate, *a* the platelet’s characteristic radius, and $G_{s}$ the in-plane surface shear modulus linked to *K*.

### 2.1. Simulation Domain and Boundary Conditions

**Figure 1:**
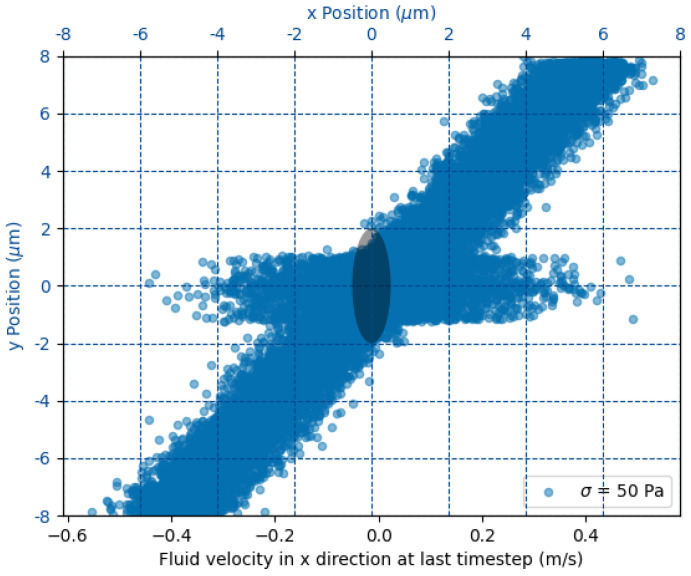
Steady-state distribution of DPD fluid particle velocities around the platelet in its initial position (dark ellipsoid at center, for reference). Each blue dot represents the x-component of a fluid particle’s velocity after relaxation, plotted against its y-coordinate, with a Couette flow of σ=50 Pa. The clear gap around the ellipsoid arises from the no-penetration boundary condition.

The goal of the simulation is to determine the dynamical evolution of a 3D ellipsoid embedded in a Couette flow. The fluid flow is described by the hydrodynamic interaction of approximately 4 million particles mediated by dissipative particle dynamics. The 3D ellipsoid is modeled by 18 thousand particles interacting via harmonic bonds characterized by a common elastic constant *K*. The computational box measures 16×16×8μm (Figure 1). Opposing translational velocities *U* are prescribed at the top and bottom walls to realize a Couette flow; this choice is motivated by the fact that, at platelet scale, more complex flow structures can be neglected, as they happen on shorter scales than the typical viscous relaxation times. The required velocity for a target shear stress σ follows the linear relation

(2) $$U\;=\;\frac{\sigma \;L}{2\mu },$$

with L=16μm the wall spacing. The velocity boundary conditions are enforced on the wall at y=±8μm via ghost particles moving with velocity *U* in opposite directions (see Figure 1). Periodic boundary conditions are enforced in the remaining directions. The time step $\Delta t=2.4\times 10^{-10}\;s$ ensures numerical stability across all $Ca^{*}$ cases, and each run is advanced for one Jeffery period, providing a complete flip-and-deform cycle of the platelet. By numerical stability, we mean that the characteristic frequency of the ellipsoid’s elastic bonds can be resolved by the selected time step in the sense of the Nyquist theorem.

Dissipative particle dynamics (DPD) provides a mesoscale framework for capturing viscous flow effects at the particle level [29]. In our implementation, the blood is modeled as DPD particles whose velocities evolve according to [28]:

(3) $$dv_{i}=\frac{1}{m}\sum_{j=1}^{N}\left(F_{c,\;ij}\;dt+F_{d,\;ij}\;dt+F_{r,\;ij}\sqrt{dt}\right)\;,$$

where $F_{c}$, $F_{d}$, and $F_{r}$ denote the conservative, dissipative, and random force contributions, respectively. The increment $dv_{i}$ is the resulting change in velocity of particle *i*. The conservative force between particles *i* and *j* depends on their separation $r_{ij}$ via

$$F_{c,\;ij}=\hat{\alpha }\;\omega_{c}\left(r_{ij}\right)\;e_{ij},\;\omega_{c}\left(r_{ij}\right)=\left\{\begin{array}{cc} 1-\frac{r_{ij}}{r_{c}}, & r_{ij}<r_{c},\\ 0, & r_{ij}\geq r_{c}, \end{array}\right.$$

with $r_{c}$ the interaction cutoff and $e_{ij}$ the unit vector from *j* to *i*. The dissipative force is defined as

$$F_{d,\;ij}=-\hat{\gamma }\;\omega_{d}\left(r_{ij}\right)\;\left(e_{ij}\;\;\cdot \;\;v_{ij}\right)\;e_{ij},$$

where $v_{ij}=v_{i}-v_{j}$. The stochastic component is

$$F_{r,\;ij}=\hat{\sigma }\;\omega_{r}\left(r_{ij}\right)\;\zeta_{ij}\;e_{ij},$$

with $\zeta_{ij}$ a zero-mean, unit-variance Gaussian random variable. Thermodynamic consistency requires $\omega_{d}=\omega_{r}^{2}$ and ${\hat{\sigma }}^{2}=2\;\hat{\gamma }\;k_{B}T$ [30]. The amplitude $\hat{\alpha }$ of the conservative force is set by $\hat{\alpha }=75\;k_{B}T/\left(\rho_{f}r_{c}\right)$ [28], where $\rho_{f}$ is the fluid particle density. After these constraints, the only remaining free parameters are the friction coefficient $\hat{\gamma }$ and the cutoff radius $r_{c}$, both calibrated in [29] for our blood-flow conditions.

### 2.2. Platelet Membrane Model

**Figure 2:**
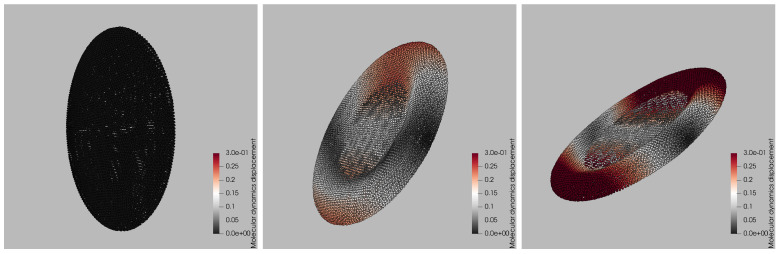
Time-resolved deformation of a platelet under Couette flow from LAMMPS simulations. Left, center, and right panels correspond to early, intermediate, and late time points within one Jeffery orbit. The color scale denotes the magnitude of each particle’s displacement vector u(t) normalized by the maximum initial displacement, i.e., $∥u\left(t\right)∥/\max_{p}∥u_{p}\left(0\right)∥$.

The platelet is initialized as a hollow ellipsoid 4×4×1μm discretized into ≈18,000 particles by 3D Delaunay triangulation. Nearest neighbors are linked by harmonic bonds,

(4) $$U_{\mathrm{harm}}=\sum_{\mathrm{bonds}}K\;{\left(r-r_{0}\right)}^{2},$$

where *K* is varied to realize the desired $Ca^{*}$ range. Non-bonded blood–platelet interactions use a truncated Lennard-Jones potential plus dissipative and random DPD forces to enforce a no-slip, non-penetrating interface [29]. The coupling between the DPD fluid and the platelet membrane is realized through a non-bonded pairwise force, giving each membrane particle an incremental velocity change:

(5) $$dv_{i}\;=\;\frac{1}{m}\sum_{j\neq i}\left(\nabla U\left(r_{ij}\right)\;dt\;+\;F_{d,\;ij}\;dt\;+\;F_{r,\;ij}\sqrt{dt}\right)\;,$$

where the conservative interaction derives from a truncated Lennard–Jones potential,

(6) $$\nabla U\left(r_{ij}\right)=4\;\varepsilon_{LJ}\left[{\left(\frac{\sigma_{LJ}}{r_{ij}}\right)}^{12}-{\left(\frac{\sigma_{LJ}}{r_{ij}}\right)}^{6}\right],$$

capturing both short-range repulsion and longer-range attraction between fluid and membrane beads. The dissipative $F_{d}$ and stochastic $F_{r}$ terms exchange momentum thermally, while the steep repulsive core enforces an effective no-slip condition at the membrane surface. All Lennard–Jones parameters $\varepsilon_{LJ}$ and $\sigma_{LJ}$ follow the calibration of Zhang et al. [29]. The resulting dynamics is depicted in Figure 2, where three representative snapshots from a single LAMMPS trajectory at $Ca^{*}=0.38$ are presented, illustrating how the platelet deforms during one Jeffery-type flip. The left panel captures the initial stage: the membrane is still close to its ellipsoid rest shape and the color map representing the magnitude of the displacement vector u(t) normalized by the maximum initial displacement, shows negligible motion. By the intermediate frame (center), the platelet has rotated about $45^{\circ }$ relative to the shear plane; viscous stresses have stretched the front and rear rims, producing the red bands (25% relative displacement) and a slight concavity in the mid-section. The late-time snapshot (right) corresponds to the end of the flip. The particle ensemble has now experienced up to 30% of the initial-diameter displacement (deep red), concentrated at the tips that momentarily align with the flow direction, while the central annulus undergoes minimal motion (gray). This spatially heterogeneous pattern characterized by large excursions near the rims and modest displacements on the flatter faces is a hallmark of shear-induced tank-treading and is reproduced consistently across all ten stiffness levels in the training set. The snapshots therefore provide a qualitative benchmark against which the surrogate’s color-by-displacement reconstructions are later compared.

For every value of *K*, we record the particle positions at each time step, yielding $O(10^{6})$ labeled states per trajectory. The resulting database underpins the DeepONet-based surrogate, which is trained on 80% subset of the trajectories and validated both *interpolatively* (within the spanned parameter grid) and *extrapolatively*, as detailed in Section 3.

## 3. Neural Operator Surrogate Model

The surrogate model is trained to emulate the operator

(7) $$G:\;\left(x_{0},\;t;\;\sigma ,\;K\right)\;⟼\;x\left(t\right),$$

where $x_{0}=\left(x_{0},y_{0},z_{0}\right)$ denotes the initial position of a membrane particle, *t* is the simulation time, σ=50 Pa is the imposed wall shear stress, and *K* the harmonic-bond elastic constant that sets the capillary number. For every membrane particle in the platelet, the network returns the particle’s instantaneous coordinates x(t)=(x(t),y(t),z(t)). Thus the surrogate learns the full, time-resolved deformation field of a platelet given its initial geometry and the governing flow–material parameters.

**Figure 3:**
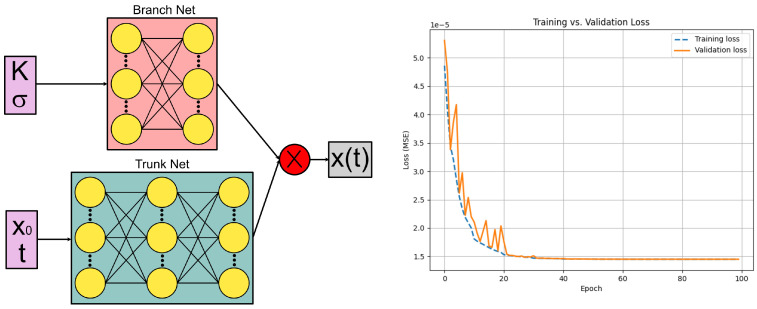
(**left panel**) Schematics of the DeepONet architecture; (**right panel**) convergence of the DeepONet: training and validation mean squared error (MSE) drop rapidly within the first ∼25 epochs, with no divergence between the two curves, indicating good generalization.

The neural network implementation (see Figure 3, left panel) follows the architecture of DeepONet [2]. Both the *branch net* and the *trunk net* are fully connected neural networks. The branch net contains 2 hidden layers with 32 and 16 nodes, while the trunk net contains 3 hidden layers with 32, 32, and 16 nodes. The latent space dimension (which is the same for both networks) is given by 32 nodes. The ReLU activation function is employed at the nodes of the hidden layers, whereas the linear activation function is reserved for the outputs. The scalars (σ,K) form the branch input, while $\left(x_{0},t\right)$ is supplied to the trunk. The output of the two network is processed via an inner product, and the corresponding output is the particle’s absolute position x(t).

The training dataset is drawn from the LAMMPS simulations (Section 2) and split 90% for training and 10% for validation. The network weights are optimized using the Adam algorithm over 100 epochs, with an initial learning rate of $10^{-3}$ that is adaptively reduced to $10^{-7}$ on plateau. The loss function is the mean squared error between the network prediction and ground truth, and we track the mean absolute error on the validation set to detect overfitting. The resulting convergence is shown in Figure 3 (right panel).

**Figure 4:**
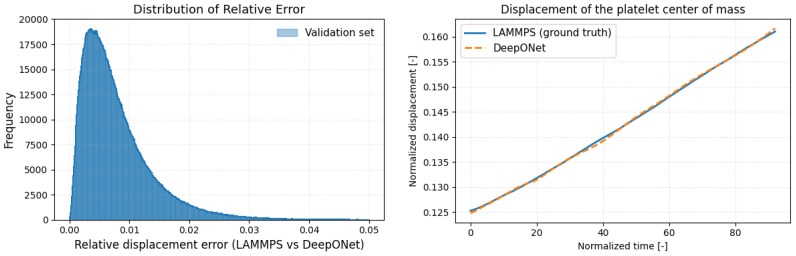
(**left panel**) Histogram of relative displacement errors on the validation set, showing that mode of DeepONet predictions incur less than 1% error relative to LAMMPS. (**right panel**) Time series of the platelet’s normalized center-of-mass displacement demonstrating near-perfect agreement between DeepONet and LAMMPS over the full time evolution.

A preliminary performance assessment compares the relative displacement error of DeepONet predictions (computed on the held-out validation set) with the corresponding LAMMPS ground truth. As shown in Figure 4 (left), the error distribution sharply peak below 1% and exhibits a long, thin tail extending to 5%. The right panel of Figure 4 demonstrates that the surrogate accurately tracks the platelet’s center-of-mass displacement throughout the Couette-flow trajectory. Achieving sub-percent error while accelerating the time-dependent deformation by several orders of magnitude highlights the surrogate’s promise for multiscale blood-flow modeling.

The DeepONet surrogate introduced above is conceived as the microscopic engine of a future multiscale hemodynamic framework. Because each prediction reduces to a single, inexpensive forward pass, the model might allow for molecular-level detail at a cost compatible with continuum CFD, offering a viable alternative to lattice-Boltzmann or immersed-boundary hybrids. Naturally, its usefulness hinges on how faithfully it reconstructs the underlying particle-dynamics trajectories: the central research question examined in this work. The following subsections outline the inference workflow and sketch, in broad terms, how the surrogate could be embedded in a continuum solver, e.g., through a force-based coupling interface to achieve platelet-resolved accuracy without resorting to prohibitively fine meshes or time steps.

### 3.1. Model Workflow

At a given time step *t*, the CFD solver provides the wall shear σ experienced by a platelet and a material-specific bond stiffness *K* (set by the target $Ca^{*}$). Those two scalars are concatenated, min–max normalized, and fed into the *branch* network. Simultaneously, each membrane particle supplies its initial coordinates $x_{0}=\left(x_{0},y_{0},z_{0}\right)$ and the dimensionless time $\hat{t}=t/T_{J}$ to the *trunk* network. The resulting 32-dimensional latent vectors b(σ,K) and $t(x_{0},\hat{t})$ are combined by an inner product,

$$\Delta x\left(t\right)\;=\;W\;\left[b\left(\sigma ,K\right)\;\cdot \;t\left(x_{0},\hat{t}\right)\right],$$

where W is a final linear layer mapping the scalar product to the three Cartesian components of the displacement. Adding Δx(t) to $x_{0}$ instantly yields the deformed node position x(t), closing the loop without time integration. During training, $\left(x_{0},\hat{t}\right)$ samples are drawn uniformly from the $\;10^{6}$ molecular dynamics states per trajectory and batched together with the corresponding (σ,K). Inference re-uses the same normalization statistics but skips gradient tracking, reducing each evaluation to ∼1 µs on a single GPU.

### 3.2. Embedding in a Continuum Solver

The surrogate is designed to interface with any finite-volume, finite-element, or lattice-based CFD solver through the following generic two-way coupling loop. A typical time step would proceed as follows:

1. The macroscopic solver evaluates the local shear or velocity gradient acting on each platelet and passes the scalar σ to the surrogate.
2. The DeepONet returns the updated membrane node positions x(t) or, equivalently, the platelet stresslet.
3. That information is converted into an effective body force or boundary traction via force-coupling, immersed-boundary, or any other particle–fluid exchange scheme and applied to the Eulerian flow field.
4. The continuum equations are advanced by the CFD solver, yielding the updated velocity field and closing the loop for the next surrogate query.

Because each surrogate evaluation is a single forward pass, the per-platelet overhead is $O(10^{-4})$ CPUs orders of magnitude smaller than the cost of the fluid solver while maintaining subplatelet-scale geometric fidelity.

### 3.3. Why DeepONet Is Advantageous

Compared with traditional reduced-order fits (POD, radial-basis interpolation) or direct coarse-grained MD–CFD coupling, the proposed operator-learning approach offers:

- **Speed**: Several orders of magnitude faster than on-the-fly particle dynamics. When embedded in a multiscale loop like the one introduced in the previous subsection, it could reasonably permit $10^{5}$ platelets in a patient-specific artery on a commodity GPU.
- **Generalizability**: Differentiable mapping across a continuous band of $Ca^{*}$; no need to store lookup tables for every stiffness/shear pair.
- **Memory efficiency**: The full surrogate weighs a few megabytes, versus terabytes for raw MD trajectories.
- **Compatibility**: The feed-forward nature of DeepONet can integrate cleanly with existing CFD codes and is amenable to adjoint or PINN-based optimization, an option not readily available with lattice-Boltzmann or immersed-boundary hybrids.

Coupling this surrogate to a continuum CFD solver provides a nanometer-resolved, data-driven alternative to low-fidelity empirical models (e.g., the Nobili damage index [31]). By directly embedding molecular-level deformation dynamics into the CFD domain, the surrogate reduces a key source of uncertainty in multiscale thrombosis predictions, potentially offering a significant long-term advantage compared to traditional approaches.

## 4. Per-Capillary Number Analysis

The aim of this section is to quantify how the surrogate error varies with membrane stiffness, expressed through the capillary number $Ca^{*}\left(\sigma ,K\right)$. A pragmatic training strategy was adopted: rather than excluding an entire stiffness level, the network was trained on a uniformly random 90% subset of $\left\{x_{0},t,\sigma ,K\right\}$ tuples, leaving the remaining 10% for validation. Consequently, every value of *K* appears in the optimizer mini-batches, but only a sparse selection of time–particle samples at that *K* influences the weights. The per-*K* study presented below evaluates the fully trained network on complete platelet trajectories, *including time instants and particle indices never seen during training*, thus providing a strict interpolation benchmark across the full $Ca^{*}$ span.

While a leave-one-*K* cross-validation would yield a pure extrapolation metric, it would also entail retraining ten separate models on datasets exceeding $10^{8}$ samples each, which is beyond the scope of the present work. The interpolation results reported here therefore represent the surrogate’s expected accuracy when embedded in multiscale hemodynamic simulations that operate within the calibrated capillary-number range.

The per-capillary-number results are summarized in Table 3, which reports the median, 90th-percentile, and maximum relative errors between DeepONet predictions and LAMMPS simulations. Relative errors increase with the capillary number (as lower bond stiffness produces larger deformations), but even in the most extreme cases the maximum error stays below 4%, demonstrating the surrogate’s high fidelity. Interestingly, the stiffest case (lowest $Ca^{*}$) shows a slightly higher maximum error than some intermediate stiffnesses. This is attributable to its location at the edge of the training domain, where the model effectively operates in a quasi-extrapolative regime. A focused assessment of extrapolation performance is presented in Section 4.

**Figure 5:**
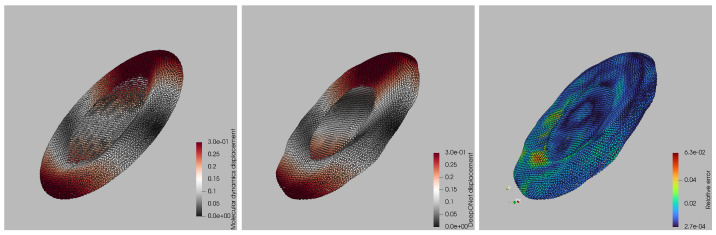
Per-particle displacement comparison at the worst-case capillary number: (**left panel**) ground-truth magnitudes from LAMMPS, (**central panel**) DeepONet-predicted magnitudes, and (**right panel**) corresponding point-wise relative error, illustrating the spatial distribution of surrogate discrepancies.

A qualitative comparison of surrogate and ground-truth displacement fields is shown in Figure 5 for the worst-case capillary number. Left and central panels display the per-particle displacement magnitudes from the LAMMPS simulation and the DeepONet prediction, respectively, using the same color scale. Visually, the two fields are almost indistinguishable: the red bands marking the leading and trailing rims, the gray mid-zone, and the subtle saddle-like depression at the platelet’s center all appear at the correct locations and amplitudes in the surrogate. The right panel maps the point-wise relative error, revealing the spatial distribution of prediction discrepancies and highlighting the regions where the surrogate deviates most from the molecular-dynamics reference. Discrepancies remain below 2% over most of the membrane and peak at 6% only in two small patches near the high-curvature tips, where the membrane briefly self-contacts. Importantly, no systematic bias is observed: the error alternates in sign and stays localized, indicating that the network has captured the global deformation modes and that residuals stem from highly localized geometric features rather than a misrepresentation of the overall dynamics. This worst-case snapshot therefore corroborates that the surrogate maintains high fidelity even under the most challenging deformation regime.

**Figure 6:**
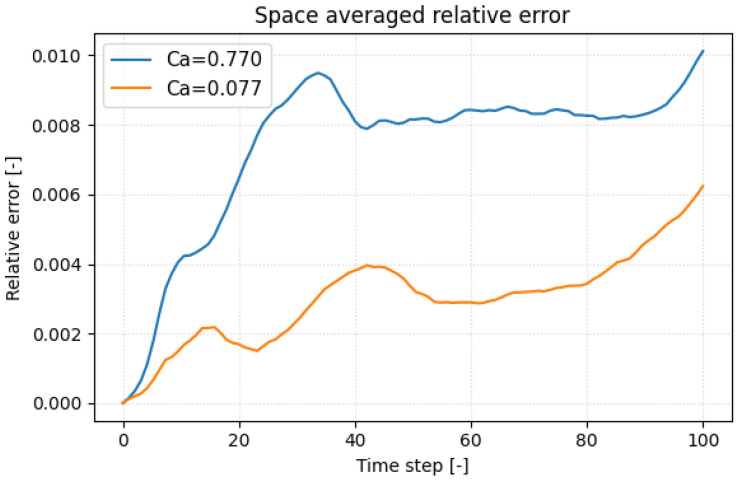
Space-averaged relative displacement error of the DeepONet surrogate versus LAMMPS ground truth over one Jeffery period for two capillary numbers ($Ca^{*}=0.77$ in blue, $Ca^{*}=0.077$ in orange). Error peaks coincide with initial membrane self-contact and rise again in the post-contact, non-periodic regime, highlighting regions of greatest model challenge.

To pinpoint when the surrogate deviates most from the molecular-dynamics reference, we plotted the space-averaged relative displacement error versus time in Figure 6 for the most compliant platelet ($Ca^{*}=0.77$) and the stiffest ($Ca^{*}=0.077$) one. In both cases the error climbs rapidly when the inner faces of the hollow membrane first touch, a kinematic discontinuity that the network finds hardest to replicate. Because the stiffer platelet deforms more slowly, this contact (and its associated error peak) occurs later in the trajectory. Such self-contact is an artifact of the hollow-shell idealization; a realistic platelet, with cytoplasm and spectrin cytoskeleton, would not experience it, so the challenge to the surrogate should lessen in future models. A secondary drift in error appears toward the end of the run, reflecting the non-periodic post-contact dynamics and the growing flow–structure complexity. Incorporating physics-informed regularization, e.g., enforcing zero net hydrodynamic torque, offers a path to reduce this late-stage error growth.

**Figure 7:**
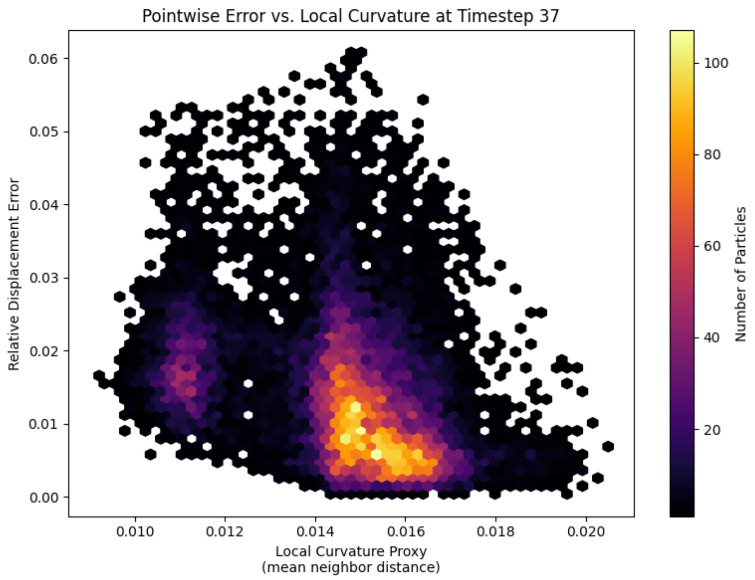
Hexagonal-bin scatter of point-wise relative displacement error versus local curvature for the most compliant platelet ($Ca^{*}=0.77$) at first self-contact (time step 37). Local curvature is proxied by the average distance to nearest neighbors, and bin color indicates the number of particles. The elevated error cluster at higher curvature values highlights the surrogate’s difficulty in capturing sharp bends and kinematic discontinuities.

At the moment of first self-contact, the spatial error distribution on the most compliant platelet ($Ca^{*}=0.77$) reveals a clear dependence on local membrane curvature (Figure 7). Each hexagon represents a patch of particles binned by its curvature proxy (mean distance to nearest neighbors) and point-wise relative displacement error. A distinct cluster at higher curvature values exhibits elevated errors, indicating that regions with tight bends and kinematic discontinuities pose the greatest challenge for the fully connected DeepONet trunk net. To better respect the platelet’s bead-spring topology and improve accuracy in these high-curvature zones, future work might replace the trunk net with a graph neural network that directly replicates the membrane graph structure.

### Extrapolation Study

**Figure 8:**
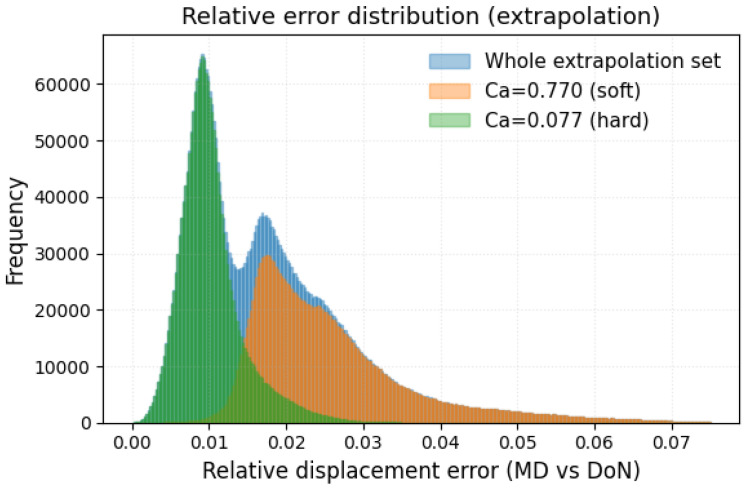
Relative displacement error histograms for the extrapolation study. The surrogate is evaluated on held-out capillary numbers: the soft-platelet case ($Ca^{*}=0.77$, orange) and the stiff-platelet case ($Ca^{*}=0.08$, green), with the combined extrapolation set in blue.

The extrapolation study trains the DeepONet surrogate model by holding out the trajectory data for the two extreme $Ca^{*}$ cases while keeping all training hyper-parameters identical to the baseline case. Performance is then evaluated solely on the excluded capillary numbers. Figure 8 shows the resulting relative error histograms: a clear bimodal distribution arises, with the lower-error peak corresponding to the stiff-platelet extrapolation (green) and the higher-error peak to the compliant-platelet extrapolation (orange). In full extrapolation mode, most particles incur errors between 1% and 3%, and the maximum error remains below 8%, underscoring the surrogate’s high-fidelity performance even outside its training envelope.

## 5. Conclusions

We developed and benchmarked a DeepONet surrogate that reproduces the full, time-resolved deformation of a platelet in shear flow at a fraction of the cost of particle-based simulation. Training on ten capillary numbers spanning $0.07\leq Ca^{*}\leq 0.77$ and $10^{6}$ particle states per trajectory, the network achieves sub-percent median errors and keeps the worst-case error below 4 % across the entire calibrated range. A leave-extreme extrapolation test confirms that accuracy degrades acceptably: 90% of the predictions for the held-out stiff and compliant platelets remain within 3% of the LAMMPS reference, and the maximum error never exceeds 8%. These results place the surrogate firmly in the high-fidelity class while delivering speed-ups of four to five orders of magnitude—opening the door to organ-scale hemodynamic simulations with clinically realistic platelet counts.

Error analysis highlights two outstanding challenges. First, localized peaks occur when the hollow membrane self-contacts, revealing the difficulty of capturing kinematic discontinuities with a fully connected trunk net. Second, a slow drift appears in the non-periodic post-contact phase. Both issues motivate future extensions: (i) replacing the trunk with a graph neural network that respects the bead–spring topology and (ii) incorporating physics-informed regularization terms (such as zero net hydrodynamic torque) to constrain long-term behavior. More realistic platelet geometries that include the cytoplasm and cytoskeleton, thereby preventing self-contact, will further reduce surrogate error. An additional input for the surrogate model that will be considered in future works is the initial orientation of the platelet with respect to the shear direction. This information plays a relevant role when computing, for example, the stresslet experienced by a platelet, and cannot be neglected in possible future coupling mechanisms with CFD solvers.

These results constitute a promising first step toward a true multiscale thrombosis framework. Key challenges remain, such as incorporating platelet–platelet interactions, vessel-wall effects, red-blood-cell collisions, and coagulation kinetics, before clot initiation and growth can be captured. In future work, we will explore the surrogate’s flexibility to address each of these components, with the ultimate goal of enabling computationally efficient, platelet-resolved hemodynamic simulations.

## Figures and Tables

**Table 1:** Examples of dimensionless capillary numbers in micro- and nano-scale blood-flow studies, related to the range considered in this work.

Application	${Ca}^{*}$ [-]	Reference
Red-blood-cell organization in straight micro-capillaries	0.07	[21]
Platelet mechanotransduction	0.2 (derived)	[22]
Red-blood-cell diffusivity and margination	0.3	[23]
Platelet–red blood cell interaction	0.7	[24]

**Table 2:** Parameter sweep defining the capillary-number dataset. The bond constant *K* is varied from 0.0003 to 0.0030 N $m^{-1}$ in steps of 0.0003 N $m^{-1}$.

Shear Stress σ [Pa]	Bond Constant *K* [N $m^{-1}$]	Resulting ${Ca}^{*}$
50	*K* = {0.0003–0.0030}	0.07–0.70

**Table 3:** Validation-set relative displacement errors for each bond stiffness *K* and corresponding capillary number $Ca^{*}$. The median (50th percentile), 90th percentile, and maximum errors of DeepONet predictions versus LAMMPS ground truth are shown.

*K* [N/m]	${Ca}^{*}$ [-]	Rel. Error (50%)	Rel. Error (90%)	Rel. Error (Max)
0.0003	0.7698	0.0094	0.0224	0.0389
0.0006	0.3849	0.0076	0.0179	0.0314
0.0009	0.2566	0.0070	0.0165	0.0288
0.0012	0.1925	0.0066	0.0158	0.0270
0.0015	0.1540	0.0062	0.0152	0.0262
0.0018	0.1283	0.0058	0.0145	0.0244
0.0021	0.1100	0.0057	0.0131	0.0212
0.0024	0.0962	0.0056	0.0122	0.0208
0.0027	0.0855	0.0058	0.0122	0.0214
0.0030	0.0770	0.0056	0.0126	0.0228

## Data Availability

The model weights and the training dataset can be provided by the author upon reasonable request.

## References

[1] Karniadakis G.E., Kevrekidis I.G., Lu L., Perdikaris P., Wang S., Yang L. (2021). Physics-informed machine learning. Nat. Rev. Phys..

[2] Lu L., Jin P., Pang G., Zhang Z., Karniadakis G.E. (2021). Learning nonlinear operators via DeepONet based on the universal approximation theorem of operators. Nat. Mach. Intell..

[3] Laudato M., Manzari L., Shukla K. (2024). High-Fidelity Description of Platelet Deformation Using a Neural Operator. arXiv.

[4] Laudato M., Manzari L., Shukla K. (2025). Neural Operator Modeling of Platelet Geometry and Stress in Shear Flow. arXiv.

[5] MacRaild M., Sarrami-Foroushani A., Lassila T., Frangi A.F. (2024). Accelerated simulation methodologies for computational vascular flow modelling. J. R. Soc. Interface.

[6] Xu Z., Chen N., Kamocka M.M., Rosen E.D., Alber M. (2008). A multiscale model of thrombus development. J. R. Soc. Interface.

[7] Tomaiuolo M., Brass L.F., Stalker T.J. (2017). Regulation of platelet activation and coagulation and its role in vascular injury and arterial thrombosis. Interv. Cardiol. Clin..

[8] Zhang J.-n., Bergeron A.L., Yu Q., Sun C., McIntire L.V., López J.A., Dong J.-f. (2002). Platelet aggregation and activation under complex patterns of shear stress. Thromb. Haemost..

[9] Zhang Y., Barocas V.H., Berceli S.A., Clancy C.E., Eckmann D.M., Garbey M., Kassab G.S., Lochner D.R., McCulloch A.D., Tran-Son-Tay R. (2016). Multi-scale modeling of the cardiovascular system: Disease development, progression, and clinical intervention. Ann. Biomed. Eng..

[10] Laudato M., Mosca R., Mihaescu M. (2023). Buckling critical pressures in collapsible tubes relevant for biomedical flows. Sci. Rep..

[11] Laudato M., Mihaescu M. (2024). Analysis of the contact critical pressure of collapsible tubes for biomedical applications. Contin. Mech. Thermodyn..

[12] Laudato M., Zea E., Sundström E., Boij S., Mihaescu M. (2024). Sound generation mechanisms in a collapsible tube. J. Acoust. Soc. Am..

[13] Gutiérrez N.G., Mukherjee D., Bar D. (2024). Decoding thrombosis through code: A review of computational models. J. Thromb. Haemost..

[14] Karmonik C., Bismuth J.X., Davies M.G., Lumsden A.B. (2008). Computational hemodynamics in the human aorta: A computational fluid dynamics study of three cases with patient-specific geometries and inflow rates. Technol. Health Care.

[15] Sundström E., Laudato M. (2023). Machine learning-based segmentation of the thoracic aorta with congenital valve disease using MRI. Bioengineering.

[16] Bornemann K.M., Jahren S.E., Obrist D. (2024). The relation between aortic morphology and transcatheter aortic heart valve thrombosis: Particle tracing and platelet activation in larger aortic roots with and without neo-sinus. Comput. Biol. Med..

[17] Zhang P., Sheriff J., Einav S., Slepian M.J., Deng Y., Bluestein D. (2021). A predictive multiscale model for simulating flow-induced platelet activation: Correlating in silico results with in vitro results. J. Biomech..

[18] Wang P., Sheriff J., Zhang P., Deng Y., Bluestein D. (2023). A multiscale model for shear-mediated platelet adhesion dynamics: Correlating in silico with in vitro results. Ann. Biomed. Eng..

[19] Gupta P., Zhang P., Sheriff J., Bluestein D., Deng Y. (2021). A multiscale model for multiple platelet aggregation in shear flow. Biomech. Model. Mechanobiol..

[20] Krüger T. (2016). Effect of tube diameter and capillary number on platelet margination and near-wall dynamics. Rheol. Acta.

[21] Liao C.T., Liu A.J., Chen Y.L. (2022). Flow-induced “waltzing” red blood cells: Microstructural reorganization and the corresponding rheological response. Sci. Adv..

[22] Abidin N.A.Z., Timofeeva M., Szydzik C., Akbaridoust F., Lav C., Marusic I., Mitchell A., Hamilton J.R., Ooi A.S., Nesbitt W.S. (2023). A microfluidic method to investigate platelet mechanotransduction under extensional strain. Res. Pract. Thromb. Haemost..

[23] Malipeddi A.R., Sarkar K. (2021). Shear-induced gradient diffusivity of a red blood cell suspension: Effects of cell dynamics from tumbling to tank-treading. Soft Matter.

[24] Vahidkhah K., Diamond S.L., Bagchi P. (2013). Hydrodynamic interaction between a platelet and an erythrocyte: Effect of erythrocyte deformability, dynamics, and wall proximity. J. Biomech. Eng..

[25] Dynar M., Ez-Zahraouy H., Misbah C., Abbasi M. (2024). Platelet margination dynamics in blood flow: The role of lift forces and red blood cells aggregation. Phys. Rev. Fluids.

[26] Tuna R., Yi W., Crespo Cruz E., Romero J., Ren Y., Guan J., Li Y., Deng Y., Bluestein D., Liu Z.L. (2024). Platelet biorheology and mechanobiology in thrombosis and hemostasis: Perspectives from multiscale computation. Int. J. Mol. Sci..

[27] Thompson A.P., Aktulga H.M., Berger R., Bolintineanu D.S., Brown W.M., Crozier P.S., In’t Veld P.J., Kohlmeyer A., Moore S.G., Nguyen T.D. (2022). LAMMPS-a flexible simulation tool for particle-based materials modeling at the atomic, meso, and continuum scales. Comput. Phys. Commun..

[28] Groot R.D., Warren P.B. (1997). Dissipative particle dynamics: Bridging the gap between atomistic and mesoscopic simulation. J. Chem. Phys..

[29] Zhang P., Gao C., Zhang N., Slepian M.J., Deng Y., Bluestein D. (2014). Multiscale particle-based modeling of flowing platelets in blood plasma using dissipative particle dynamics and coarse grained molecular dynamics. Cell. Mol. Bioeng..

[30] Espanol P., Warren P. (1995). Statistical mechanics of dissipative particle dynamics. Europhys. Lett..

[31] Nobili M., Sheriff J., Morbiducci U., Redaelli A., Bluestein D. (2008). Platelet activation due to hemodynamic shear stresses: Damage accumulation model and comparison to in vitro measurements. ASAIO J..

